# Corrosion behavior and cytocompatibility of fluoride-incorporated plasma electrolytic oxidation coating on biodegradable AZ31 alloy

**DOI:** 10.1093/rb/rbw036

**Published:** 2016-10-26

**Authors:** Peng Tian, Feng Peng, Donghui Wang, Xuanyong Liu

**Affiliations:** State Key Laboratory of High Performance Ceramics and Superfine Microstructure, Shanghai Institute of Ceramics, Chinese Academy of Sciences, Shanghai, 200050, P.R. China

**Keywords:** Mg alloy, fluoride, corrosion resistance, cytocompatibility

## Abstract

Fluoride-incorporated plasma electrolytic oxidation (PEO) coating was fabricated on biodegradable AZ31 alloy. The surface morphologies and phases were investigated by scanning electron microscopy and X-ray diffraction. The effect of fluoride incorporation in coatings on corrosion behaviour was investigated in simulated body fluid and *in vitro* cytocompatibility of the coatings was also studied by evaluating cytotoxicity, adhesion, proliferation and live–dead stain of osteoblast cells (MC3T3-E1). Furthermore, the corrosion morphologies *in vivo* were examined. The results showed that the fluoride could be incorporated into the coating to form MgF_2_ phase. *In vitro and in vivo* degradation tests revealed that the corrosion resistance of the coating could be improved by the incorporation of fluoride, which may attribute to the chemical stability of MgF_2_ phase. Moreover, good cytocompatibility of fluoride-incorporated coating was confirmed with no obvious cytotoxicity, enhanced cell adhesion and proliferation. However, when the fluoride content was high, a slight inhibition of cell growth was observed. The results indicate that although fluoride incorporation can enhance the corrosion resistance of the coatings, thus resulting a more suitable environment for cells, the high content of fluoride in the coating also kill cells ascribed to the high released of fluorine. If the content of fluoride is well controlled, the PEO coating with MgF_2_ phase is a promising surface modification of Mg alloys.

## Introduction

Metal-based materials have been widely used in orthopedic implants and bone fixtures, which require proper mechanical strength to support the growth of injured tissues [1–3]. However, these non-biodegradable materials, such as stainless steel, cobalt–chromium alloys and titanium alloys, will still remain in human body for a long period of time after tissues healing. The long existence of these implants may cause lasting irritations or harms to the surrounding tissues because of the abrasion debris [4] or toxic metal ions [5] released from the implants. Moreover, the elastic modulus of these non-biodegradable metals usually shows obvious difference with that of natural bone, leading to a mismatch at the interface between implant and surrounding tissue, which usually causes stress shielding showing side effect on the tissues reconstruction [6]. As biodegradable in physiological environment, magnesium-based materials have attracted much attention as the substitutes of the above-mentioned non-biodegradable metals due to their good biocompatibility and suitable mechanical properties, especially a match in elastic modulus with the natural bone [7]. With the complete degradation of magnesium-based implants, a second surgery will be avoided after the injured tissues healed [8]. Nevertheless, owing to their high chemical reactivity, the magnesium-based implants will degrade rapidly *in vivo*, which results in fast loss of their mechanical strength and some adverse effects such as local alkalization, hydrogen evolution around the implants [7, 8]. So the control of corrosion rate of magnesium-based implants is not only critical for retaining the mechanical integrity of the implants but also beneficial for reducing adverse effects caused by the fast degradation of the implants.

To enhance the corrosion resistance of the magnesium-based implants, fabricating a coating on magnesium substrate is feasible [9]. Besides chemical conversion coatings [10, 11] and degradable polymer coatings [12, 13], plasma electrolytic oxidation (PEO) coatings have been widely investigated because of their high adhesion strength to the substrate and their adjustable compositions [14]. In physiological environment, as magnesium fluoride is more chemically stable than magnesium oxide, the corrosion resistance of magnesium substrate could be enhanced by increasing the F/O ratio of the surface coating [15]. The addition of fluoride into the PEO coatings to form magnesium fluoride could effectively improve their corrosion resistance [16, 17]. As coatings for biomedical applications, the safety and functions of the added ions are necessary to be considered. The previous investigations show that the fluoride ions are beneficial for prevention of the dental caries as they can concentrate in enamel and dentine [18, 19]. However, the excess of fluoride will induce toxicity [20, 21]. So when enhancing the corrosion resistance of the PEO coatings by fluoride addition, their cytocompatibility should be taken into consideration and the proper content of fluoride in the coating should be determined.

In the present study, fluoride was used as addition to control the corrosion resistance of the PEO coatings. The corrosion behaviours and cytocompatibility of AZ31 magnesium alloys with different fluoride-containing coatings were evaluated *in vitro*.

## Materials and methods

### Substrate material and coatings fabrication

The AZ31 magnesium alloy with a size of 10 mm × 10 mm × 2 mm was prepared by mechanical cutting. Before the process of PEO, samples were grounded with 1000# SiC paper and then ultrasonically cleaned with acetone and ethyl alcohol.

The coatings were fabricated on AZ31 alloy by PEO equipment (Pulsetech, China). In the process of PEO, the constant current density was 50 mA/cm^2^, frequency was 300 Hz and duty cycle was 10%. The process was last for 15 min. After that, the coated samples were boiled in de-ionized water for 10 min to remove the remained electrolytes. The fluoride-free coating was prepared in basic electrolyte containing 0.04 M Na_2_SiO_3_·9H_2_O and 0.1 M KOH (sample obtained was denoted as PEO). Fluoride-incorporated coatings were obtained in basic electrolytes with 0.05 M, 0.1 M and 0.2 M KF·2H_2_O as fluoride source (samples obtained were denoted as PEOF0.05, PEOF0.1 and PEOF0.2, respectively).

### Coatings characterization

The surface morphologies of all coatings were characterized by scanning electron microscopy (SEM; Hitachi-S3400N, Hitachi, Japan), and elemental compositions of the samples surfaces were detected by energy dispersive spectrometry (EDS, IXRF-550i, IXRF SYSTEMS, USA). The crystalline phases of all coatings were identified by X-ray diffraction (XRD; Diffractometer D8, Bruker, Germany).

### *In vitro* and *in vivo* corrosion behaviour evaluation

#### Electrochemical test

Potentiodynamic polarization test was conducted to evaluate the corrosion resistance of AZ31 alloy and coating samples by a CHI760C electrochemical analyzer (Shanghai, China) with a saturated calomel electrode as reference electrode, a graphite rod as counter electrode and a sample tested area was 0.255 cm^2^ as working electrode. The electrolyte was simulated body fluid (SBF) [22] with the temperature of 37 °C. The open circuit potential was conducted with time of 30 min, and the scanning rate was 1 mV/s. The CHI760C software was used to calculate the corrosion potential (*E*_corr_), corrosion current density (*i*_corr_) and corrosion resistance (R_p_) according to Tafel extrapolation.

#### Local alkalization test

The pH values of SBF immersion solution were measured to evaluate the alkalization degree of SBF immersion solution caused by the degradation of the samples. Samples were placed inside a 24-well cell culture plate, and each well contained 2-ml SBF with the temperature of 37 °C. After 1 day, the pH value of the solution taken out from the culture plate was measured. Then, the well was filled with 2-ml fresh PBS and its pH value was detected intermittently every day until 28 days later.

#### Hydrogen evolution test

The hydrogen released with the degradation of AZ31 alloy, PEO and PEOF0.2 in SBF at 37 °C was collected through a custom-designed hydrogen evolution set up. The volume of collected hydrogen was calculated through recording the water level intermittently every day for up to 28 days. Each group had three samples, and the exposure area of the sample to SBF volume was 10 cm^2^/L. The corrosion rate of tested samples can be figured out according to the following equation [23]:

(1) $$r=\frac{PV}{RT}\times \frac{M}{At}$$

where *r* is the corrosion rate (mg·cm ^−^ ^2^·d ^−^ ^1^), *P* is standard atmospheric pressure (Pa), *V* is volume of H_2_ (ml), *R* is 8.314 J/(mol·K), *T* is the temperature (K), *M* is the molar mass (g/mol), *A* is the exposure surface area (cm^2^) and *t* is the experiment time (day).

#### SBF immersion test

The coating samples were immersed in SBF at 37 °C for 1, 7 and 28 days to evaluate their *in vitro* corrosion behaviour and bioactivity. At the scheduled time, the samples were rinsed with distilled water and then dried in air. After that, the surface morphologies of the samples were observed by SEM, and surface elemental compositions were detected by EDS.

#### *In vivo* corrosion test

To evaluate corrosion behaviour of the samples *in vivo*, the subcutaneous implantation of animals were conducted. Mice used in the experiment were donated by Shanghai No. 6 People's Hospital. Before the test, the mice were anesthetized with pentobarbital. After shaved and disinfected, one subcutaneous pocket was made on the back of each mouse. The samples were implanted in the pockets and were removed at 20 days after implantation. After cleaned with distilled water and dried in air, the surface morphologies of the samples were observed with SEM.

### *In vitro* biocompatibility evaluation

#### Cell culture

The osteoblast-like cells MC3T3-E1 were obtained from Cells Resource Center of Shanghai Institute for Biological Science. Cells were cultured in the media with suppliers in a humidified atmosphere of 5% CO_2_ at 37 °C and were passaged every 3 days.

#### Cytotoxicity evaluation

After sterilized by ultraviolet irradiation for 12 h, the samples were cultured in culture medium for 24 h. The ratio of sample area to extraction medium was 0.5 cm^2^/ml. The extracts was named as 100% and diluted to 60% and 30% before utilization. Meanwhile, cells were seeded on a 96-well culture plated with a density of 1 × 10^4^ cell per well. After cultured for 24 h, the culture medium was removed, and 200-μl extracts with different concentrations were added. One and 4 days later, the cytotoxicity of the samples was evaluated by 3-(4,5)-dimethylthiahiazo (-z-y1)-3,5-di- phenytetrazoliumromide (MTT) assay. Fresh culture medium without any extract and 5% Dimethyl sulfoxide (DMSO)-containing culture medium severed as the negative control and the positive control, respectively. According to the instruction of the MTT assay, the absorbance of the formazan solutions was determined by an enzyme-labeling instrument (BIO-TEK, ELX 800) at 492 nm, and the cells viability was calculated as follows:

(2) $$Viability=\frac{{AS}_{492}-{AP}_{492}}{{AN}_{492}-{AP}_{492}}\times 100\%$$

where AS_492_ is the absorption value of the samples, AP_492_ is the absorption value of the positive control and AN_492_ is the absorption value of the negative control.

#### Cell adhesion evaluation

AZ31 alloy and coating samples were placed in 24-well culture plates. Then 1.5-ml cell suspension with a density of 7.0 × 10^4^ cell/ml was seed on the specimens. After incubation for 1, 4 and 24 h, the cells were gently rinsed with PBS three times. Then the cells were fixed, permeabilized and blocked with 4% paraformaldehyde solution, 0.1% (v/v) Triton X-100 and 1 wt% BSA solution, respectively. Subsequently, the cells were stained with Fluorescein Isothiocyanate (FITC)-Phalloidin and further staining with 2-(4-Amidinophenyl)-6-indolecarbamidine dihydrochloride (DAPI, Sigma, USA). The cytoskeletal actin and cell nuclei were characterized with a fluorescence microscopy (Olympus, Japan).

#### Cell proliferation evaluation

Cell suspension of 1.5 ml with a density of 5.0 × 10^4^ cell/ml was seeded on the specimens. The cells were measured after 1- and 4-day cultivation according to the following steps. The samples were cleaned with PBS twice and then 1-ml fresh complete α-MEM medium containing 5 v% MTT replaced the previous culture medium. Four hours later, the culture media were replaced with 1-ml DMSO to dissolve the formazan. Solutions of 100 μl for each sample were transferred to a 96-well culture plate, and the absorbance was measured at 492 nm.

#### Live/dead cell staining

The live–dead cell staining kit (Biovision, USA) was performed according to manufacturer’s instructions. Briefly, cells were cultured on the specimens with a density of 5 × 10^4^ cells per well. Propidium iodide and calcium-AM were diluted to final concentrations of 5 and 2 μM in PBS, respectively. After 4 days, 100 μl of mixed solution was added to each specimen, and the cells were stained at 37 °C for 15 min.

#### Hemolysis ratio

Human whole blood was obtained from Zhongshan Hospital of Shanghai. Blood (0.8 ml) was diluted by 1 ml 0.9 wt% NaCl aqueous solution. After rinsed with 0.9 wt% NaCl aqueous solution, each sample was extracted in 1.5 ml 0.9 wt% NaCl aqueous solutions for 30 min at 37 °C. Untreated 0.9 wt% NaCl aqueous solution and distilled water served as negative and positive controls, respectively. Then, 30-μl diluted blood was added to the samples and incubated for 60 min. Then, the solutions were centrifuged for 5 min at 3000 rpm. The supernatant was measured at 545 nm, and hemolysis ratio (HR) was calculated as follows:

(3) $$HR=\frac{\;{AS}_{545}-{AN}_{545}}{\;{AP}_{545}-{AN}_{545}}\times 100\%$$

where AS_545_ is the absorption value of the samples, AP_545_ is the absorption value of the positive control, and AN_545_ is the absorption value of the negative control.

### Statistical analysis

All statistical analysis was performed using a GraphPad Prism 5 statistical software package. All of the data are expressed as the mean ±  SD. Statistically significant differences (*P*) between the various groups were measured using two-way Analysis of Variance (ANOVA).

## Results and discussion

### Coating characterization

**Figure 1:**
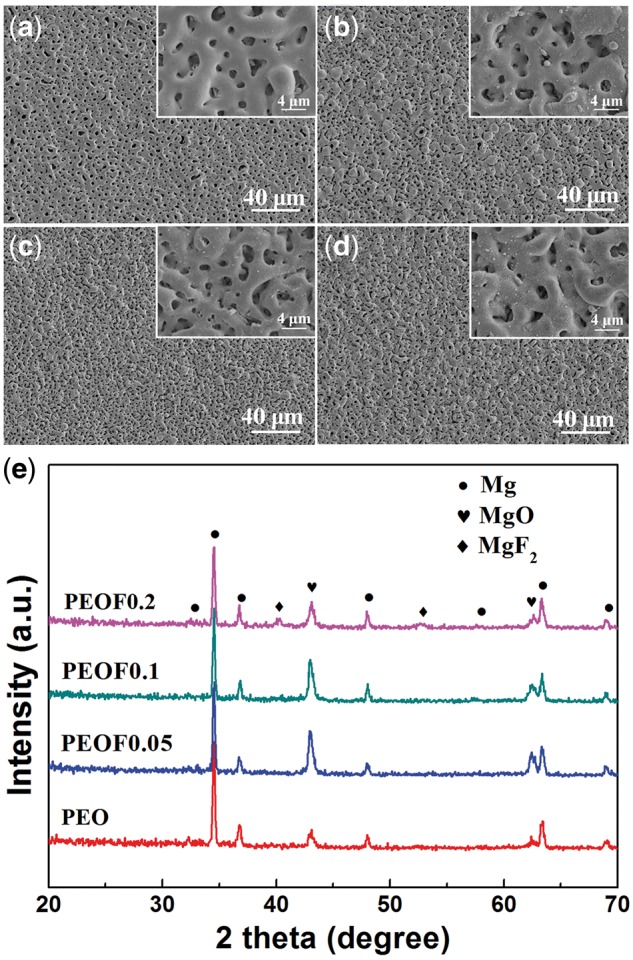
Surface morphologies of PEO (**a**), PEOF0.05 (**b**), PEOF0.1 (**c**), PEOF0.2 (**d**) coatings and XRD patterns of all coating samples (**e**).

The surface morphologies of coating samples are shown in Fig. 1a–d. It can be clearly seen that all the coatings showed porous surface structure. These pores form by the molten oxide and gas emission out of the micro-arc discharge channels during the PEO process. Compared with PEO (Fig. 1a), PEOF0.05 (Fig. 1b), PEOF0.1 (Fig. 1c) and PEOF0.2 (Fig. 1d) showed rougher surface morphologies. With the increase of fluoride content in the coating, the amount of pores increased while the average diameter of pores decreased. The change of pore structures may be contributed to the various energy conditions of micro-arc discharge in different electrolytes.

The elemental compositions of the coating samples are summarized in Table 1. Apart from Mg, Al and Zn from the magnesium substrate, the PEO coating also contained O and Si, which came from the electrolyte during the PEO process. C is also detected attributing to the contamination on the samples surface in the air. With the increase of fluoride content in the electrolyte, the O, Si decreased and F increased in the formed coating.

**Table 1:** Surface elemental compositions of the coating samples

Samples	Elements (At.%)						
	Mg	Al	Zn	C	O	Si	F
PEO	24.91	1.04	0.22	14.67	51.26	7.90	–
PEOF0.05	28.12	1.30	0.26	13.80	49.47	5.20	1.85
PEOF0.1	31.03	1.39	0.12	10.28	47.55	4.91	4.72
PEOF0.2	26.74	0.68	0.37	12.90	41.56	4.25	13.50

The XRD patterns of coating samples are shown in Fig. 1e. In the pattern of PEO, feature peaks of MgO appeared besides of the feature peaks of Mg from the magnesium substrate, indicating the formation of crystallized MgO in the coating. When the concentration of fluoride in the coating was low, as shown in the patterns of PEOF0.05 and PEOF0.1, no significant difference of feature peaks was observed compared with that of PEO. However, when the fluoride content was comparatively high, feature peaks of MgF_2_ was observed, as shown in the pattern of PEOF0.2, suggesting the formation of MgF_2_ in the coating. In the PEO process, the ions, especially negative charged ones, in the electrolyte can take part in the formation of the coatings and react with magnesium to form compounds. In the basic electrolyte, the main ions comprised ${\text{SiO}}_{3}^{2-}$ and OH^-^, which could react with magnesium as O resource to form MgO. When fluoride ions appeared in the electrolyte, they could react with magnesium to form more stable phase MgF_2_. Because MgF_2_ is more stable than MgO in physiological environment, the formation of MgF_2_ phase by fluoride incorporation in coating is anticipated to enhance its corrosion resistance.

### *In vitro* and *in vivo* corrosion behaviors

#### Potentiodynamic polarization curves

**Figure 2:**
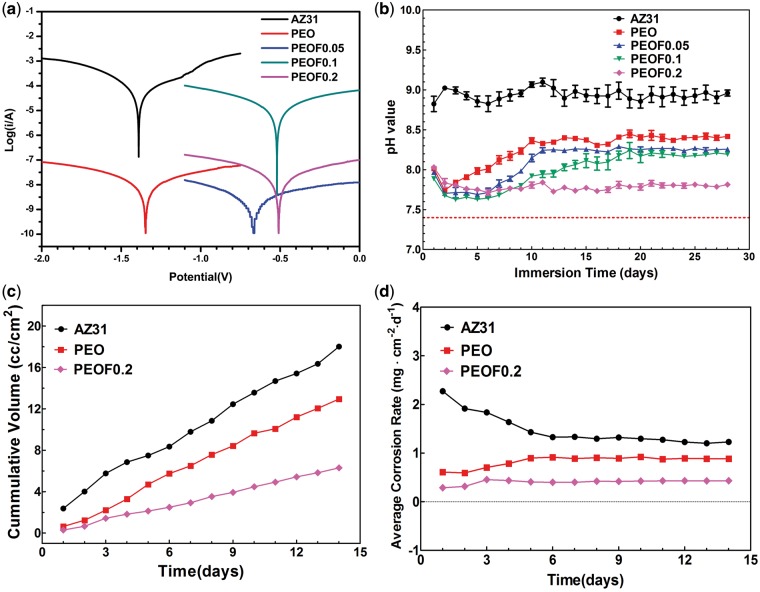
Polarization curves (**a**) and pH value changes (**b**) (data are presented as the mean ± SD, *n* = 3) of all samples, hydrogen evolution (**c**) and corrosion rate (**d**) of AZ31 alloy, PEO and PEOF0.2 immersed in SBF.

Figure 2a presents polarization curves of all the samples, and Table 2 summarizes the corrosion potential (*E*_corr_), corrosion current density (*i*_corr_) and corrosion resistance (*R*_p_). Compared with AZ31 alloy, the *E*_corr_ value of PEO presented no obvious difference, while its *i*_corr_ value was decreased with about four orders of magnitude and its *R_p_* value was increased with about four orders of magnitude. Compared with PEO, after fluoride incorporation in the coating, the potentiodynamic curves of PEOF0.05, PEOF0.1 and PEOF0.2 presented obvious changes with more positive *E*_corr_. This change may be attributed to the change of surface pore structure and phase composition.

**Table 2:** Corrosion potential (e_corr_), corrosion current density (i_corr_) and corrosion resistance (rp) calculated according to the polarization curves

Samples	Ecorr (V)	icorr (A/cm^2^)	Rp (Ω)
AZ31	−1.383	1.686 × 10^−4^	6.606 × 10^3^
PEO	−1.347	2.233 × 10^−8^	4.626 × 10^7^
PEOF0.05	−0.667	6.149 × 10^−9^	1.742 × 10^8^
PEOF0.1	−0.517	2.793 × 10^−5^	3.599 × 10^4^
PEOF0.2	−0.508	3.150 × 10^−8^	3.545 × 10^7^

#### pH values

Figrue 2b shows the pH changes of the SBF solutions. It revealed that all samples resulted in an increase of pH value due to their alkaline degradation products. Compared with AZ31 alloy, all the coatings showed good protection for the magnesium substrate, especially at the initial immersion stage. However, when the immersion time extended, the protection effectiveness of the coatings presented significant difference, showing that the higher the fluoride content was in the coating, the longer the protection time the coating presented. During the test period of 28 days, the pH value change of SBF solution containing PEOF0.2 sample was comparatively stable and much smaller than that of AZ31 alloy and other coating samples.

#### Hydrogen evolution curves and corrosion rates

Hydrogen evolution by the rapid degradation of magnesium-based implant *in vivo* will lead to a gas cavity around the implant and cause damage to tissue due to the insufficient absorbance or transportation of the released hydrogen through surrounding tissues [8]. The AZ31 alloy, PEO and PEOF0.2 were picked out as typical samples and the hydrogen evolution from them in a period up to 14 days are shown in Fig. 2c. As anticipated, the AZ31 alloy presented a fast release of hydrogen, while the PEO sample exhibited smaller volume of released hydrogen. After fluoride incorporation, the PEOF0.2 coating showed better protection for the substrate with smaller volume of released hydrogen compared with AZ31 and PEO. As the release of hydrogen is resulted from the degradation of magnesium substrate, the corrosion rate of the magnesium substrate with or without coatings can be approximately calculated according to the hydrogen evolution data and the result is shown in Fig. 2d. It can be concluded that the AZ31 alloy corroded quickly, especially at the initial immersion stage, and decreased with time extended, which may be ascribed to the corrosion products formed on its surface. Besides, the corrosion rate of PEO sample was lower than AZ31 alloy. The magnesium substrate under PEOF0.2 coating was best protected, showing the lowest corrosion rate.

#### Surface morphologies and elemental compositions after corrosion

**Figure 3:**
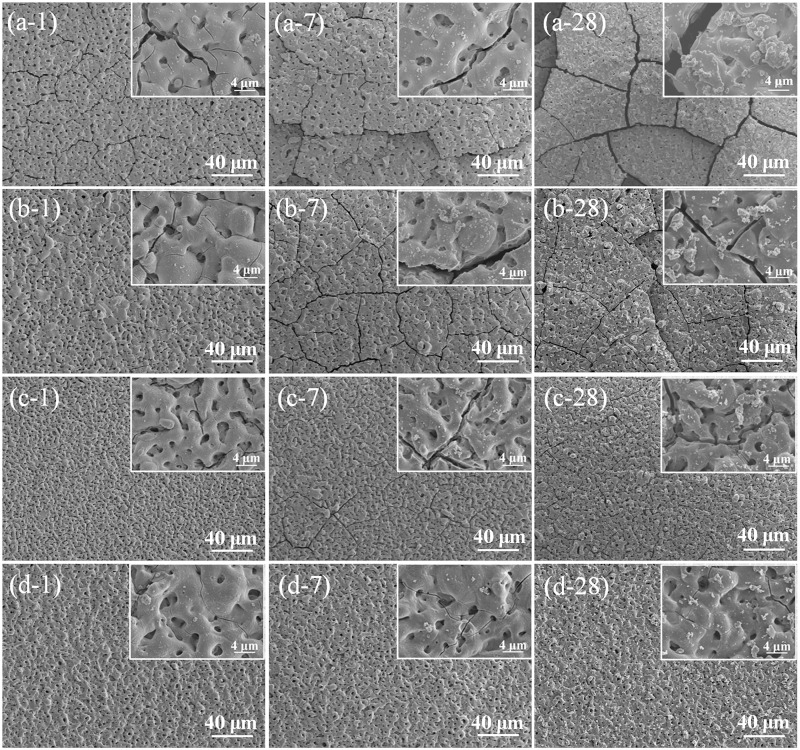
Surface morphologies of PEO (**a**), PEOF0.05 (**b**), PEOF0.1 (**c**) and PEOF0.2 (**d**) after immersed in SBF for 1 (i-1), 7 (i-7) and 28 (i-28) days (i stands for a, b, c and d).

**Figure 4:**
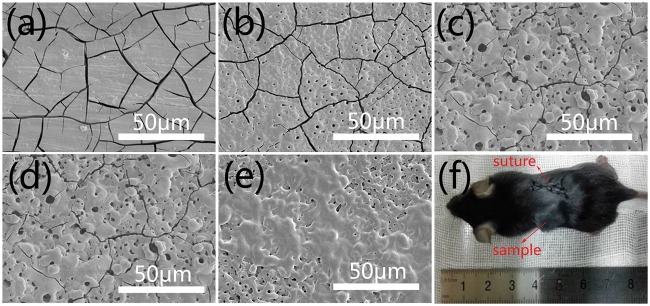
Surface morphologies of AZ31 (**a**), PEO (**b**), PEOF0.05 (**c**), PEOF0.1 (**d**) and PEOF0.2 (**e**) after implanted in mice for 20 days and image of the mouse after implantation (**f**).

**Figure 5:**
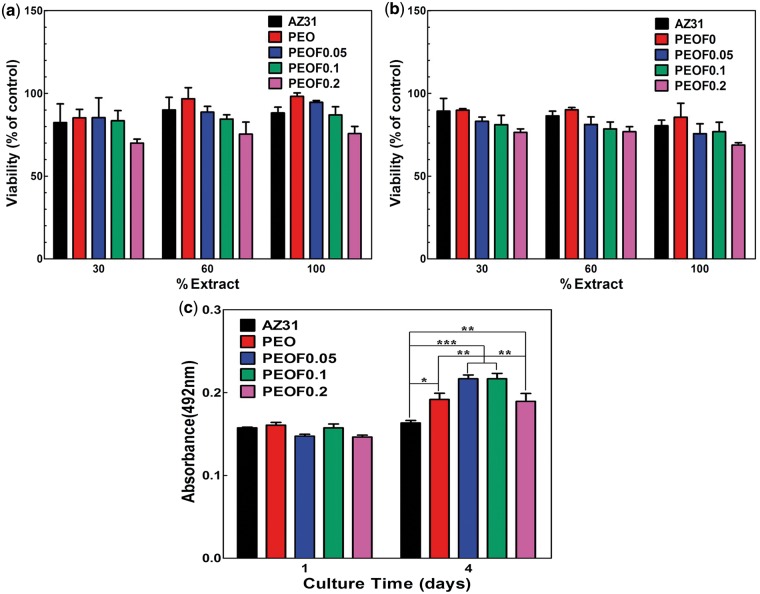
Viability of MC3T3-E1 cells incubated for 1 day (**a**) and 4 days (**b**) with different concentration extracts of AZ31 alloy and coating samples, and proliferation of MC3T3-E1 cells cultured on AZ31 alloy and coating samples for 1 day and 4 days (data are presented as the mean ± SD, *n* = 3, and analyzed using a two-way ANOVA, *P < 0.05, **P < 0.01, ***P < 0.001).

**Figure 6:**
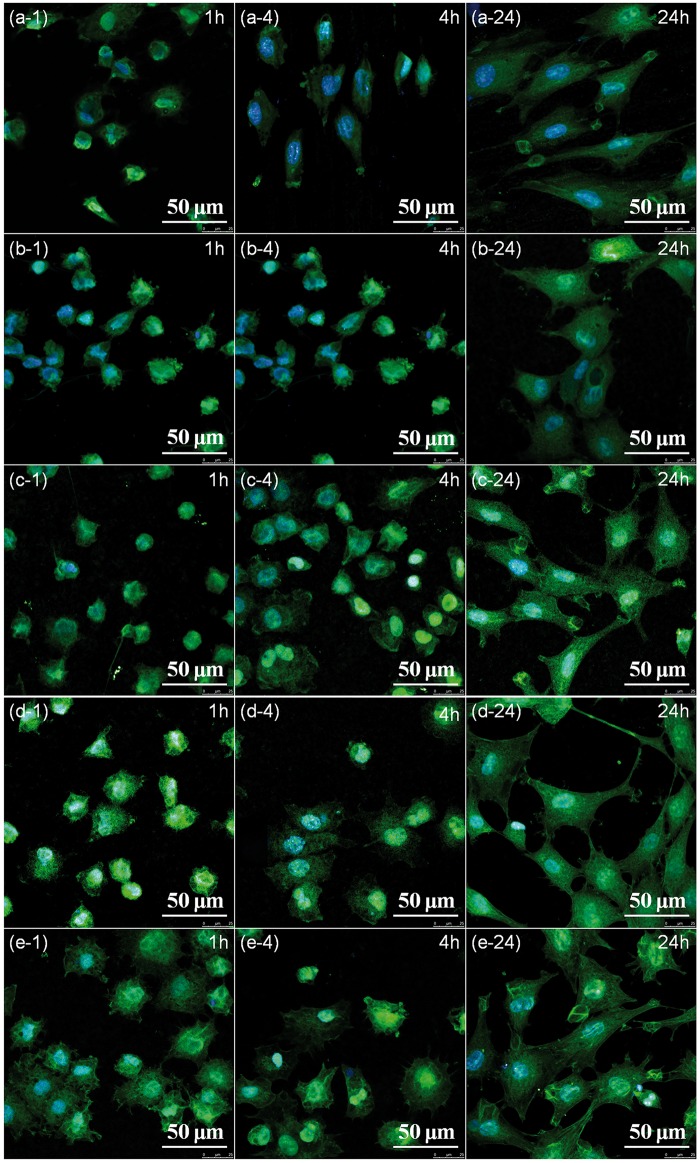
Fluorescence microscopy images of MC3T3-E1 cells cultured on AZ31 alloy (**a**), PEO (**b**), PEOF0.05 (**c**), PEOF0.1 (**d**) and PEOF0.2 (**e**) for 1 h (i-1), 4 h (i-4) and 24 h (i-24) (i stands for a, b, c and d) with actin stained with FITC (green) and the nucleus stained with DAPI (blue). Full color version available online.

**Figure 7:**
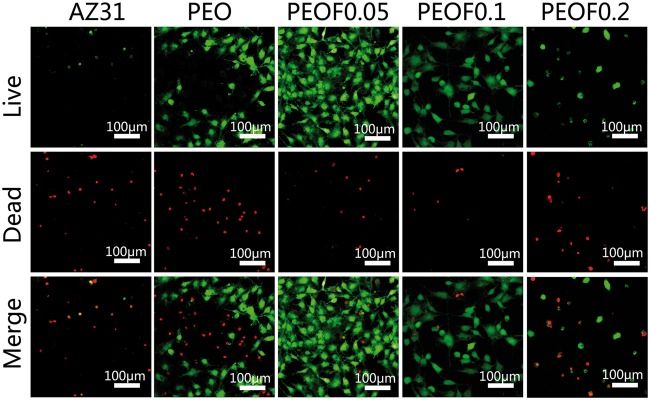
The CLSM images of live/dead staining of MC3T3-E1 cells after 4 days of culturing on various surfaces.

In order to observe the failing mechanism of the coatings, the surface morphologies of coating samples immersed in SBF are shown in Fig. 3. After immersion for 1 day, the PEO started to corrode and many cracks appeared on the surface (Fig. 3a-1). For magnesium-based materials, magnesium react with water in physiological environment will form a film mainly composed of Mg(OH)_2_, which can connect with H_2_O molecules and form Mg(OH)_2_·*n*H_2_O hydrate. After dried, dehydration of hydrate will cause film shrinks and then cracks appear. The cracks formed on PEO can be attributed to the partial degradation of coating and magnesium substrate, indicating that the more cracks appeared, the more corrosion products formed. When fluoride was incorporated into the coating, the corrosion resistance of coating was significantly enhanced. No obvious cracks were observed on the surfaces of PEOF0.05, PEOF0.1 and PEOF0.2 after 1-day immersion (Fig. 3b-1, c-1, d-1). When extended to 7 days, the cracks on PEO became longer and deeper (Fig. 3a-7), while cracks appeared on PEOF0.05 (Fig. 3b-7) and PEOF0.1 (Fig. 3c-7). The surface integrity of PEOF0.2 was still remained with no obvious cracks observed (Fig. 3d-7). After 28-day immersion, the PEO corroded severely with complete broken surface structure (Fig. 3a-28), and some obvious cracks were clearly observed on PEOF0.05 (Fig. 3b-28) and PEOF0.1 (Fig. 3c-28). Even after immersion for 28 days, the surface morphology of PEOF0.2 was not obviously changed only with some corrosion particles and micro-cracks distributing on its surface (Fig. 3d-28).

The corresponding elemental compositions of all coating samples after immersion are summarized in Table 3. Compared with the elemental composition before immersion, the Mg content decreased and the O content increased on PEO coating after immersion, resulting in a value change of O/Mg ratio from 2.05 to 4.66. This change can be attributed to the partial degradation of MgO in the coating and magnesium substrate to form Mg(OH)_2_. Similar to the trend revealed on PEO, the decrease of Mg and increase of O were also detected on PEOF0.05, PEOF0.1 and PEOF0.2. When immersed in SBF for 28 days, the final values of O/Mg ratio for PEOF0.05, PEOF0.1 and PEOF0.2 were 4.01, 3.5 and 2.43, respectively. As the value of O/Mg ratio can be considered as an indicator of degradation degree and more degradation will induce a bigger O/Mg ratio value, the final O/Mg ratio values also reveal that the fluoride can stabilize the coating to reduce its degradation rate and the PEOF0.2 coating has the best corrosion resistance. Moreover, the partial degradation of all samples to increase the pH value of immersion solution induced the deposition of Ca-P deposits with a small amount of Ca and P on the sample surface detected.

**Table 3:** Surface elemental compositions of coating samples immersed in SBF for 1, 7 and 28 days

Immersion time (days)	Samples	Elements (at. %)								
		Mg	Al	Zn	C	O	Si	F	P	Ca
1	PEO	11.81	1.21	0.18	14.32	54.24	9.50	–	6.42	2.32
	PEOF0.05	16.14	1.49	0.29	14.85	51.58	6.66	2.01	5.09	1.91
	PEOF0.1	13.96	1.68	0.34	13.70	51.20	6.85	4.83	5.25	2.19
	PEOF0.2	18.83	1.21	0.39	14.21	44.01	3.91	9.63	5.56	2.25
7	PEO	11.35	1.63	0.21	10.85	57.71	9.98	–	5.85	2.42
	PEOF0.05	10.01	2.40	0.28	10.16	57.95	9.24	2.53	4.87	2.57
	PEOF0.1	13.06	2.10	0.24	8.23	55.94	7.28	5.84	4.82	2.50
	PEOF0.2	19.20	1.55	0.26	9.29	46.93	4.51	11.84	4.31	2.11
28	PEO	11.52	1.45	0.23	10.12	59.57	7.85	–	6.05	3.21
	PEOF0.05	15.78	1.29	0.33	11.34	63.34	3.72	0.30	2.75	1.15
	PEOF0.1	16.51	1.91	0.34	5.50	57.82	6.13	5.17	4.61	2.01
	PEOF0.2	21.07	1.91	0.31	4.77	51.33	4.52	10.75	3.70	1.64

#### *In vivo* corrosion

Owing to *in vitro* corrosion environment is different from that *in vivo*, it is essential to evaluate the corrosion resistance of the samples *in vivo*. Figure 4 displays the corrosion morphologies of the samples after implanted in mice for 20 days and the image of the mouse after implantation (Fig. 4f). It is clear that less cracks on the surface of PEO (Fig. 4b) coating than bare AZ31 alloy (Fig. 4a), and after incorporated with fluoride (Fig. 4c, d and e), the amount of cracks were further decreased. It is noticeable that few cracks on the surface of PEOF0.2 (Fig. 4e) and some of the pores forming during the PEO process were covered. Fewer cracks meant better corrosion resistance, and the result was consistent with *in vitro* corrosion test. The matters covered on the pores of PEOF0.2 might be cells or tissues tightly adhered to the sample according to the result of *in vitro* cell adhesion (Fig. 6).

### *In vitro* biocompatibility

#### Cytotoxicity

Evaluation of cytotoxicity is critical for a new biomaterial. Figure 5a and b shows the viability of MC3T3-E1 cells cultured in different concentration extracts of all specimen. After 1-day incubation (Fig. 5a), compared with AZ31 alloy, the viability of cells in PEO extract was higher, which may be attributed to less alkalization (Fig. 2b) and ions released from the coating, such as Si ions [24, 25]. The viability of cells in extracts of PEOF0.05, PEOF0.1 and PEOF0.2 decreased to some extent, but still remained above 70%, showing no obvious cytotoxicity. When it came to 4 days, all samples showed no obvious cytotoxicity and the same trend was revealed that the excess fluoride in the coating could inhibit the cell growth, which is completely in accordance with the previous study [26].

#### Cell proliferation on various surfaces

Implant-tissue osteointegration and the reconstruction of the injured tissue need a fast cell proliferation on the surface of implant. Figure 5c shows the proliferation of MC3T3-E1 cells on AZ31 alloy and coating samples. After culture for 1 day, no significant difference was observed on various surfaces. When extended to 4 days, compared with AZ31 alloy, cells proliferate significantly faster on coating samples. Besides, the cells grew even faster on PEOF0.05 and PEOF0.1 than on PEO. However, a slight inhibition of cell growth was observed on PEOF0.2. This result can be explained with stability of coating structure and fluoride ions release. When the fluoride content in the coating increases, the corrosion resistance is enhanced with more stable structure and little influence on the change of cell culture media, which is beneficial for the cell growth. Moreover, the proper amount of fluoride ions released from fluoride-containing coating improves the growth of cells [26, 27]. However, the excess amount of fluoride ions may be released from PEOF0.2 inhibiting the cell growth as the cells response differently with different concentration fluoride in the culture media [26].

#### Cell adhesion and spread on various surfaces

The good adhesion of cells is critical for their following growth and proliferation on the implants. Figure 6 shows the cytoskeletons of MC3T3-E1 cells at the initial adhesion and spreading process. The cells on AZ31 alloy showed a round shape after 1-hour incubation (Fig. 6a-1). The cells on PEO, PEOF0.05 and PEOF0.1 were also not spread (Fig. 6b-1, c-1, d-1), while the expression of filamentous F-actin for cells on PEOF0.2 was better (Fig. 6e-1). After incubation for 4 hours, the cells preferred to adhesion and spread on the coatings, especially on the fluoride containing ones (Fig. 6c-4, d-4, e-4), compared with AZ31 alloy (Fig. 6a-4), showing more expression of filamentous F-actin. After incubation for 24 hours, this trend became more significant (Fig. 6i-24). The cells on PEOF0.2 (Fig. 6e-24) showed better spreading than the other samples with development of filopodia and lamellipodia. The previous study shows that the cell adhesion process of cells consist of substrate attachment, spreading, cytoskeleton development, survival and then proliferation [28]. Therefore, favorable adhesion is a critical step for the integration between tissue and implant. The enhanced process of cell adhesion on the fluoride-containing coating may be attributed to the stabilization of the coating structure by fluoride addition.

#### Live/dead cell staining

Live/dead staining was conducted to further investigate the effect of the samples to the MC3T3-E1 cells, and the result is shown in Fig. 7. Compared with AZ31 alloy, the dead cells on the PEO coating were almost the same, while the live cells were significantly increased. When a small quantity of fluoride was incorporated into PEO coating, the amount of live cells was further increased and that of dead cells was decreased. However, once the fluoride was too much, as PEOF0.1 and PEOF0.2 coatings, it would be disadvantage to live cells. The phenomenon can be explained by the same reason mentioned above. Briefly, cells grow better on a surface with better corrosion resistance and can be improved with a small amount of fluoride, but inhibited when there is too much fluoride released.

#### Hemolysis ratio

Blood-contacting materials demand a low HR. Higher HR means the material will cause greater harm to the erythrocyte cells. The HRs of the samples are summarized in Table 4. The HR of bare AZ31 alloy was very high, but dramatically decreased after PEO processes. It was mainly ascribed to the better corrosion resistance of PEO coating than AZ31.After fluoride incorporated, though the HR was slightly increased, it was still much lower than 5% which is the safe level for blood-contacting materials according to the ISO 10993-4. Surface structure and fluoride released from the coatings might play a role in HR. As the content of fluoride increased, the size of pores decreased and more fluoride released into the physiological environment, thus resulting a higher HR.

**Table 4:** HR of different samples (data are presented as the mean ± SD, *n* = 4)

Sample	AZ31	PEO	PEOF0.05	PEOF0.1	PEOF0.2
HR (%)	52.94	0.65	0.92	0.78	2.48

## Conclusions

The fluoride-incorporated coating is prepared on AZ31 alloy by PEO process. The coating was mainly composed of MgO and MgF_2_. The fluoride incorporation in the coating enhanced its corrosion resistance *in vitro* and *in vivo*. Less local alkalization and hydrogen evolution were observed with the coating protection. The enhanced corrosion resistance of the fluoride-incorporated coating may be attributed to its chemical stability in physiological environment with MgF_2_ phase as stabilizer. The fluoride incorporation did not induce obvious cytotoxicity, and cell adhesion was enhanced on the coating surface. The coating with low fluoride content also improved the cell growth on its surface while the coating with high fluoride content induced a slight inhibition of cell growth. Although the fluoride incorporation is feasible to enhance the corrosion resistance of the coating fabricated by PEO process, high fluoride content in the coating will induce inhibition of cell growth. So the proper fluoride content in the coating should be carefully determined to meet a balance between good corrosion resistance and cytocompatibility.
