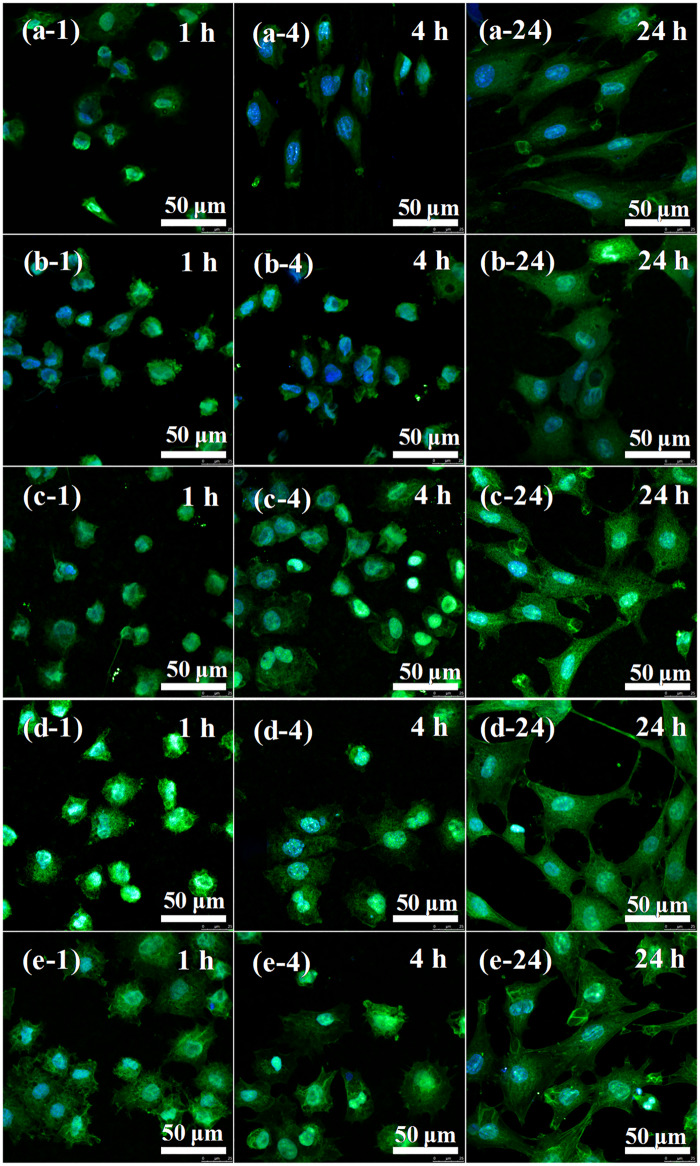# Corrigendum to: Corrosion behavior and cytocompatibility of fluoride-incorporated plasma electrolytic oxidation coating on biodegradable AZ31 alloy

**DOI:** 10.1093/rb/rbaa016

**Published:** 2020-06-03

**Authors:** Peng Tian, Feng Peng, Donghui Wang, Xuanyong Liu

**Affiliations:** State Key Laboratory of High Performance Ceramics and Superfine Microstructure, Shanghai Institute of Ceramics, Chinese Academy of Sciences, Shanghai, 200050, P.R. China

doi: 10.1093/rb/rbw036, *Regenerative Biomaterials* 2017;4:1–10

Figure 6 of this article contained an accidental duplication in the fluorescence images of MC3T3-E1 cells cultured on PEO group. A second fluorescece image showing cell cultures after 4 hours (Fig. 6 b-2) was incorrectly shown in the place of cell cultures after 1 hour (Fig. 6 b-1). A corrected version of the figure appears below.

**Figure 6:**